# Assessing Progress Towards SDGs Implementation Using Multiple Reference Point Based Multicriteria Methods: The Case Study of the European Countries

**DOI:** 10.1007/s11205-022-02886-w

**Published:** 2022-01-31

**Authors:** E. Ricciolini, L. Rocchi, M. Cardinali, L. Paolotti, F. Ruiz, J. M. Cabello, A. Boggia

**Affiliations:** 1 University of Perugia, Perugia, Italy; 2 Programa de Doctorado en Economía y Empresa, Universidad de Málaga, Málaga, Spain; 3 University of Málaga, Málaga, Spain

**Keywords:** Sustainable development goals, Multicriteria analysis, Weak-strong composite indicators, Multi reference point based partially compensatory indicator, 2030 Agenda

## Abstract

To achieve the UN 2030 Agenda Goals, and considering their complexity and multidisciplinary, Multi-criteria analysis appears to be a suitable approach to give a true support to public decision makers in defining policy lines. This study focuses on the application of the Multiple Reference Point Weak-Strong Composite Indicators (MRP-WSCI) and its partially compensatory version (MRP-PCI), to assess, in the framework of the UN 2030 Agenda, the sustainability of the 28 members of the European Union (pre-Brexit). Countries were analyzed and compared according to their conditions and progress against the 17 Sustainable Development Goals, considering three reference years: 2007, 2012 and 2017. The analysis shows that Nordic countries reach a good level of global sustainability, with values of the indicators, W-W-W and S-W-W, between 2 and 3; while the States of east Europe, in particular Romania, Bulgaria and Greece, stay at the worst levels, having overall indicators values less than 1.5. Furthermore, the results highlight how countries in the lower group have difficulties especially in social and economic sustainability. On the other hand, states with a good overall condition record the worst results in the environmental dimension, such as the Netherlands, which shows, for the year 2017, a value for this sphere less than 2, while in the other two show a good value (over 2.5).

## Introduction

The 2030 Agenda is an action plan designed for the prosperity of people and the planet, established in 2015 by the United Nations. In the current economic, political, and social context, the Agenda promotes global peace and the eradication of poverty in all its forms and dimensions. It represents the current world’s greatest challenge. In addition, it is an essential requirement for reaching a true sustainable development (United Nations, 2015). All countries and stakeholders, acting in collaborative partnership, are mobilised to implement this plan by 2030. The Agenda is based on 17 Sustainable Development Goals (SDGs), which follow and expand the prior United Nations Millennium Development Goals (MDGs). The latter ended in 2015 and represented the most significant global effort to deal with sustainable development in a comprehensive way. Developed by UN member nations, the 17 goals, organised into 169 targets, identify global development priorities, effectively defining sustainable development through the three pillars: economic, environmental and social (Stevens et al., 2016).

According to the UN Agenda 2030 and the Sustainable Development Goals, Policy Makers must consider the sustainability perspective in strategic planning decisions. Identifying and measuring the level of sustainability, through its three dimensions, is a priority (Gusmão Caiado et al., 2018). This is the only way to understand if and how a Community, a Region, a Nation is following the sustainability path. To assess whether, and to what extent, the SDGs are reached, Decision Makers need suitable technical support, to conduct ex-ante, in progress monitoring and ex post evaluations of the advancement towards sustainability. Decision support systems provide appropriate information to improve the decision-making process itself, selecting and measuring the various activities that contribute to sustainable development (Scrase et al., 2002).

Although the importance of assessing the progress to reach SDGs is recognised, the academic literature about possible approaches is limited (Gusmão Caiado et al., 2018). In particular, a new framework is needed to address the barriers and challenges that the SDGs pose. Establishing clear target values within the Sustainable development objective context is complicated by the broad scope of the goals themselves, by the various scales (national, regional, global), by the coverage of multiple issues in a single objective, and by ambiguous language (Allen et al., 2019).

McArthur and Rasmussen (2019) applied to Canada the assessment of the progress towards each goal at national level, where data collection is easier, using the philosophy of “no one left behind.” They first filtered out targets that are not outcome-focused at the country level. They then analysed the data availability. However, at national level quantification is not always possible for all the targets and so they adopted a logic to integrate the missing ones, based on a proxy-approach which allowed them to overcome the problem. Also, Allen et al. (2020) assessed the progress of a developed country (Australia) across some of the SDG targets. They also highlighted, through a brief review, that despite the growing importance of said indicator-based progress assessments, there is still limited academic literature available addressing and evaluating the approaches and methods applied. Schmidt-Traub et al. (2017) aggregated indicators within each country for each goal. The method does not weigh up the relative importance of the single SDGs. This is consistent with UN member states’ intentions, that framed the SDGs as an “integrated and indivisible” agenda, whereby the goals have equal priority. However, the method checks for the availability of data across SDGs in each country, favouring nations which fulfil them.

Firoiu et al. (2019) used dynamic analysis methods and prediction tools in order to assess the achievement of the SDGs by Romania. They analysed Eurostat data pertaining to the period 2007–2017 and they then calculated individual dynamic indices for each indicator. In particular, they considered the possibility of a point of convergence between the evolution trend of indicators for Romania and the one recorded for the EU average in the year 2030.

Some methods to evaluate SDGs objectives can be found in Bidarbakhtnia (2020), who showed the three major approaches taken by Sustainable Development Solutions Network (SDSN), United Nations Economic and Social Commission for Asia and the Pacific (UNESCAP) and Organisation for Economic Co-operation and Development (OECD), proposing to use one or another, depending on criteria availability. The approaches used by OECD and SDSN are comparable, measuring the distance of each indicator from the 2030 objective, for each country, and presenting it as a value-sharing ratio between similar countries (using a standard deviation to normalise the distance value). The result is a ranking for both methods, based on a normalised score on a scale of 0–10. In addition, the OECD approach identifies the direction of change by estimating the Spearman correlation coefficient between time and indicator values. A positive correlation therefore describes that a country/region is progressing in the right direction to reach the target, while a negative correlation means that it needs a course correction to reach the 2030 targets. The UNESCAP method shows also progress made by each indicator since 2000 in proportion to the total progress needed for the region to reach the 2030 objective; moreover, it determines the distance from the 2030 targets as a percentage of the total importance that the region must carry out between 2015 and 2030.

The selection of appropriate indicators can be very ambiguous without an accurate and scientific follow up on their operationalisation. The process should thus be undertaken within a conceptual framework and a clear reference context. Experts should primarily focus on the “indicator-indicated fact” relation to ensure the SDGs indicators relevance (Hák et al., 2016). Moreover, according to Lancker and Nijkamp (2000), ‘a given indicator does not say anything about sustainability, unless a reference value such as thresholds is given to it’.

Given these circumstances, composite indicators (CI) appear a suitable method. The CI should ideally measure multidimensional concepts that cannot be complemented by a single indicator, providing simple comparisons that can be used to illustrate complex issues and different sectors, such as the economy, society, environment or technological development (Fusco et al., 2018). The construction of composite indicators is therefore one way of managing large amounts of information, obtained from the set of individual indicators. The aggregation of different indicators into a single synthetic measure always implies a certain loss of information on the way. The aggregation thus has to be as informative as possible, so that the results obtained can be easily and efficiently interpreted. In this way, the information contained in the composite indicator will achieve their maximum explanatory power (Ruiz et al., 2020; El Gibari et al., 2021).

In the case of the SDGs, given the large number of objectives, targets and indicators, it remains difficult to make sense of an evaluation and communicate the results without some form of aggregation, both at the objective level and through a selection of objectives and indicators, or at national level for all objectives and indicators. Some applications of a CI to SDGs are present in the literature, though there are few.

The problem of determining the most appropriate way of aggregating indicators is found in the use of partial indicators to construct an index, as shown by Marti and Puertas (2020). Their study proposes the use of the data envelopment analysis (DEA) method as a tool for aggregating the Sustainable Society Index (SSI), avoiding any prior aggregation of the categories that comprise the index. It is a powerful tool applicable to multidimensional studies, and it is not affected by the subjectivity associated with the allocation of weightings. DEA is based on two fundamental hypotheses. Firstly, all the Decision-Making Units (DMUs) in the sample are functionally similar -i.e., they all have the same type and number of inputs and outputs. Secondly, the set of DMUs has to be homogeneous, in other words directly comparable. Cluster analysis is recommended to ensure compliance with the assumptions. This is a very limiting factor especially for the measurement of sustainability, as it is a heterogeneous concept.

An attempt to use composite indicators for measuring Agenda 2030 goals can be found in Delli Paoli and Addeo (2019). They built a composite indicator for each SDG (goal index). Then, they aggregated goal indices in the three Pillars of Sustainability (Pillar Indexes) and used them to build an overall SDG index to assess European Member States performances. One of the major limitations of Paoli and Addeo’s study is the use of only the latest available data for each indicator: it does not consider historical data, since the availability of such time series is too limited for some indicators. As a result, the goal indexes, the pillar indexes and the overall SDG tell us where a country currently stands on each of the considered indicators. However, they cannot be used to infer the speed at which a country is moving towards the achievement of SDGs.

Considering all these aspects, the aim of the present paper is to assess the sustainability of the European Union countries, in particular by referring to the SDGs and using composite indicators. As previously discussed, sustainability assessment is preferably carried out by considering reference levels (thresholds, targets, etc.). On the other hand, the compensability issue is critical when measuring sustainability. Fully compensatory schemes allow a bad performance in one indicator to be offset by good performances in others, while partially compensatory or non-compensatory schemes limit this possibility. This distinction is in line with the concepts of weak and strong sustainability. While fully compensatory schemes provide an overall assessment of the sustainability of a given territory, non-compensatory methods help to pinpoint weak points of the territory and therefore, possible improvement areas. For these reasons, we have chosen to use the multicriteria analysis methods Multiple Reference Point Weak-Strong Composite Indicators (MRP-WSCI) and Multiple Reference Point based Partially Compensatory Indicator (MRP-PCI), (Ruiz & Cabello, 2021; Ruiz et al., 2020). As previously mentioned, the main advantages of these schemes are twofold. Firstly, they allow the use of reference levels for each indicator, which makes it possible to use absolute or relative benchmarks and to express the results in terms of the position of each country with respect to them. Secondly, they produce composite indicators with different degrees of compensation. On the one hand, the joint consideration of the Weak and Strong MRP-WSCI composite indicators allow decision makers not only to assess the global performance of a country, but also to find out possible improvement areas. On the other hand, the partially compensatory MRP-PCI composite indicator provides an overall measure, where the use can decide the compensation level of each element considered in the study. As already seen, multicriteria-based composite indicators are widely used in literature for analysing ever-increasing amounts of information (El Gibari et al., 2019). Different versions of these schemes have already been used to build composite sustainability indicators in Ruiz et al. (2011), Cabello et al., (2014, 2019, 2021).

Based on the literature analysed, it can be said that the process of individuation and measurement of progress toward a sustainable development is a worldwide key issue. In this paper MRP-WSCI and WRP-PCI are used for the sustainability assessment of the 28 members of the European Union (pre-Brexit). Sustainable development is a core principle of the Treaty on European Union and a priority objective for the Union’s internal and external policies and so a great commitment for the whole Union.

The countries have been analysed and compared according to their own conditions and progress, with reference to the 17 Sustainable Development Goals (SDGs) of 2030 Agenda, considering their evolution over three reference years: 2007, 2012 and 2017. In comparison to the previous literature, we included the policy dimension, through the use of weightings deriving from European strategic documents. Moreover, the method allows us to have both an evaluation for the three pillars separately and an overall one. Additionally, the time dimension has been included by considering a 10-year data horizon, one of the aspects missing in previous studies.

## Materials and Methods

### MRP-WSCI and MRP-PCI

Let’s set out the basic ideas of the reference point methodology used to build weak and strong (MRP-WSCI, Ruiz et al., 2020) and partially compensatory (MRP-PCI, Ruiz & Cabello, 2021) composite indicators. To this end, let us assume that we wish to aggregate a set of *I* indicators, for *J* regions under analysis. Let *a*_*ij*_ denote the value of indicator *i* in region *j*. This methodology assumes that a set of reference levels are given for each indicator. In this paper, we will assume that, for each indicator *i*, these statistical levels (referring to the values of all the units considered) are ${q}_{i}^{0}$ (minimum level), ${q}_{i}^{1}$ (percentile 25), ${q}_{i}^{2}$ (percentile 50), ${q}_{i}^{3}$ (percentile 75) and ${q}_{i}^{4}$ (maximum level). In this way, we are interested in studying the relative position of each region for each indicator, with respect to these reference levels, which inform us about the position of each unit with respect to the rest of them. Besides, it will be assumed that there are weights ${\omega }_{i}$ assigned to each indicator, so that

$$\sum_{i=1}^{I}{\omega }_{i}=1$$

Making use of this information, the first step consists of using a so-called achievement function, which measures the position of each region with respect to the reference levels, and at the same time, it brings all the indicators down to a common scale (0-1-2-3-4). Depending on the degree of compensation of the composite indicator, the following functions are used (assuming that the indicator has a the-more-the better direction):

- Fully compensatory (Weak) scheme. The achievement function is defined as follows:
  $${s}_{ij}^{w}=t-1+\frac{1}{{q}_{i}^{t}-{q}_{i}^{t-1}}({a}_{ij}-{q}_{i}^{t-1})$$, if $${a}_{ij}\in \left[{q}_{i}^{t-1},{q}_{i}^{t}\right]$$, *t* = 1,…,4
- Non-compensatory (Strong) scheme. The weights are normalised in the following way:
  $${\omega }_{i}^{s}=\frac{{\omega }_{i}}{\underset{k=1,\dots ,I}{\mathit{max}}\left\{{\omega }_{k}\right\}}$$
  and the achievement function is built as follows:
  $${s}_{ij}^{s}=t+\left({s}_{ij}^{w}-t\right){\omega }_{i}^{s}$$, if $${s}_{ij}^{w}\in (\left.t-1,t\right]$$, *t* = 1,…,4
- Partially compensatory scheme. In this case, we also assume that a compensation degree ${\lambda }_{i}\in [\mathrm{0,1}]$ is given for each indicator. Then, a given indicator *i* and unit *j*, if we denote ${I}_{ij}=\left\{k\in \left\{1,\dots ,I\right\}/{s}_{kj}^{w}\ge {s}_{ij}^{w}\right\}$, we define its fully compensated value:
  $${c}_{ij}=\frac{{\sum }_{k\in {I}_{ij}}{\omega }_{k}{s}_{kj}^{w}}{{\sum }_{k\in {I}_{ij}}{\omega }_{k}}$$
  and the corresponding partially compensatory achievement function is:
  $${s}_{ij}^{p}={s}_{ij}^{w}+{\lambda }_{i}\left({c}_{ij}-{s}_{ij}^{w}\right)$$
  Let us briefly explain the rationale behind each one of these functions. The achievement function ${s}_{ij}^{w}$ is a piecewise linear function that assumes values between *t*-1 and *t* if the corresponding indicator assumes values between ${q}_{i}^{t-1}$ and ${q}_{i}^{t}$ for region *j*. The non-compensatory function ${s}_{ij}^{s}$ is equal to ${s}_{ij}^{w}$ if *i* is the highest weighted indicator (${\omega }_{i}^{s}$=1), and its value is improved as the weight of the indicator decreases (but always staying in the corresponding interval). In this way, bad values in not highly weighted indicators are regarded as less bad. Finally, the fully compensated value ${c}_{ij}$ contains the value of ${s}_{ij}^{w}$, compensated by all other indicators that perform better to equal to indicator *i* for unit *j* and the partially compensatory achievement function ${s}_{ij}^{p}$ is between the original value of ${s}_{ij}^{w}$ (when the compensation degree ${\lambda }_{i}$ is 0) and this fully compensated value (when the compensation degree ${\lambda }_{i}$ is 1).
  Once the achievement functions are defined, the different composite indictors are built as follows:
- Fully compensatory (Weak) composite indicator of region j:
  $${W}_{j}=\sum_{i=1}^{I}{\omega }_{i}{s}_{ij}^{w}$$
- Non-compensatory (Strong) composite indicator of region j:
  $${S}_{j}=\underset{i=1,\dots ,I}{\mathrm{min}}\left\{{s}_{ij}^{s}\right\}$$
- Partially compensatory composite indicator of region j:
  $${PCI}_{j}=\underset{i=1,\dots ,I}{\mathrm{min}}\left\{{s}_{ij}^{p}\right\}$$

${W}_{j}$ measures the overall performance of region *j*. ${S}_{j}$ points out the worst indicator of region *j* (relativized by the corresponding weights). For example, if ${S}_{j}>1$, this means that all the indicators are over ${q}_{i}^{1}$ (percentile 25). In particular, ${S}_{j}=0$ when the region gets the worst possible value (${q}_{i}^{0}$, minimum) in the highest weighted indicator. Finally, ${PCI}_{j}$ is the minimum partially compensated achievement. Hence, each indicator is compensated according to its compensation degree ${\lambda }_{i}$. In the extreme cases, if ${\lambda }_{i}=0$ for all the indicators, ${PCI}_{j}={S}_{j}$ and if ${\lambda }_{i}=1$ for all the indicators, ${PCI}_{j}={W}_{j}$.

**Fig. 1:**
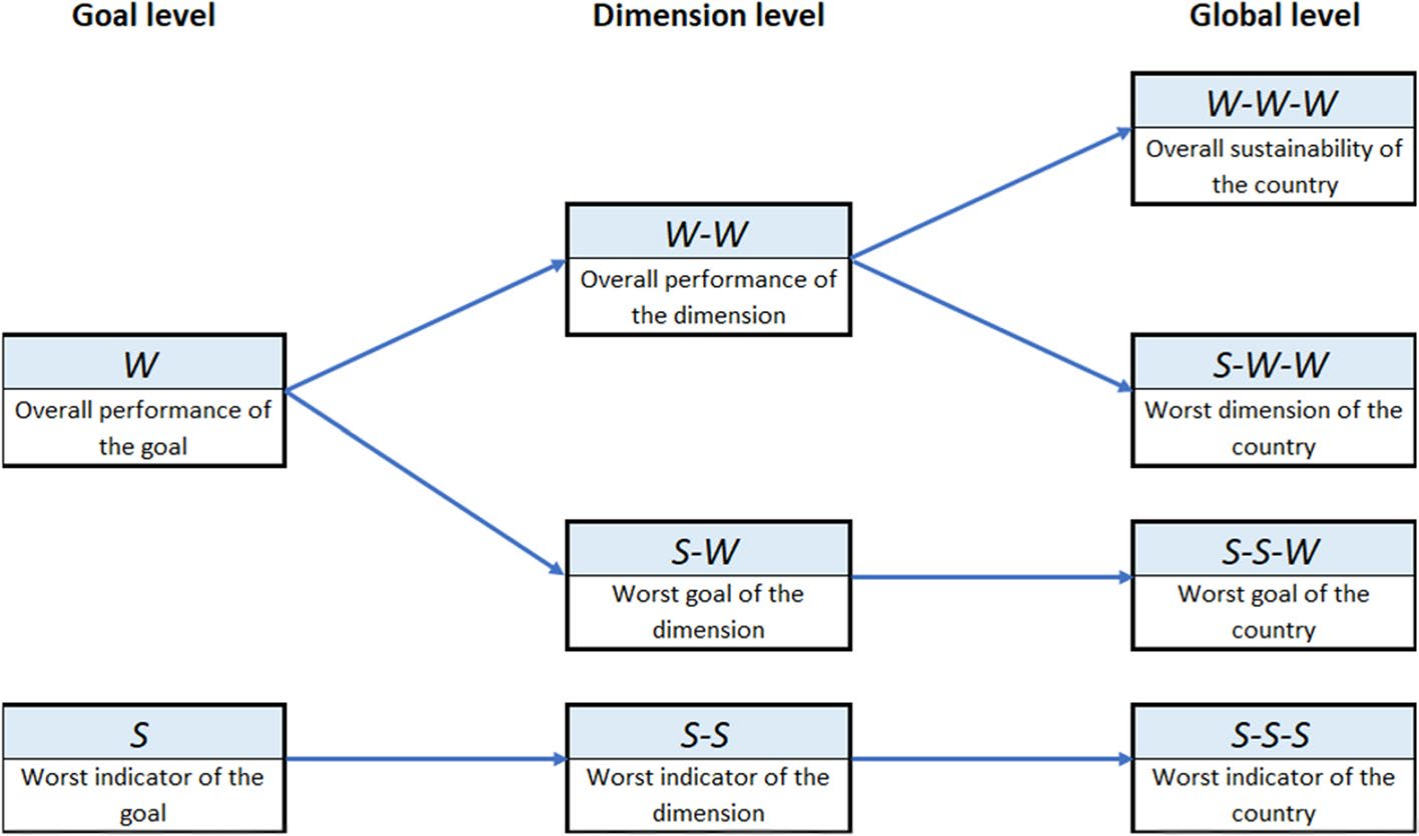
Relevant composite indicators obtained at each aggregation level using MRP-WSCI methodology

By definition, all the composite indicators obtained take values on the same scale (0-1-2-3-4) as the achievement functions, and they can thus be interpreted as the global position of the region with respect to the reference levels. Besides, there are frequently several aggregation levels in a problem. In the case of this paper, there are three levels. Firstly, the single indicators of each goal are aggregated. Secondly, the goals of the same dimension are aggregated. Finally, the three dimensions are aggregated. For these successive aggregations, the composite indicators obtained in the previous one can be used as achievement functions in the previous expressions. For the case of the MRP-WSCI methodology, several relevant composite indicators can be obtained at each level (see Fig. 1). In the case of the MRP-PCI methodology, a single composite indicator is obtained at each level.

### Case Study

**Fig. 2:**
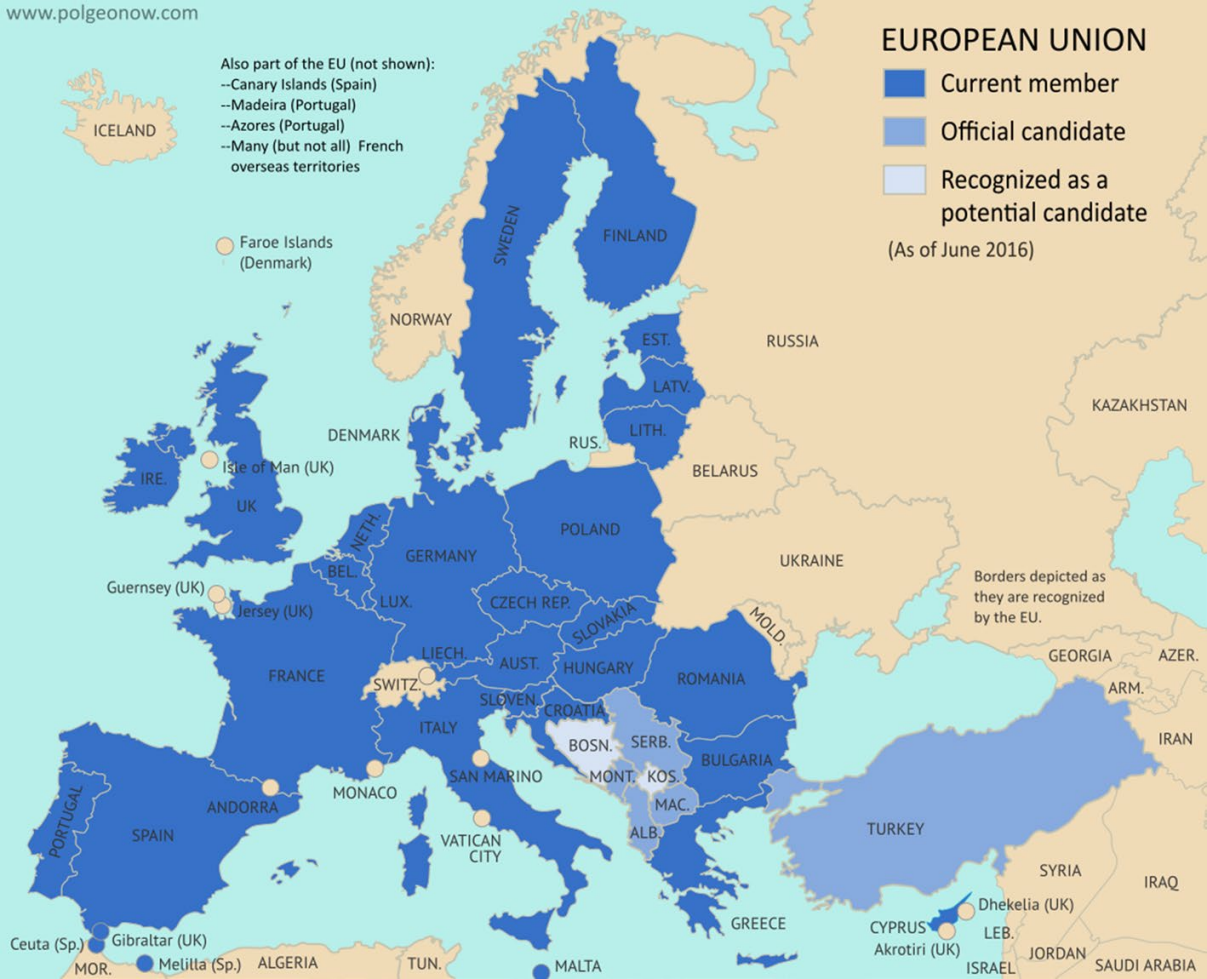
UE-28 Countries. Source: https://europa.eu/european-union/about-eu/

The purpose of this paper is to conduct a comparative sustainability analysis among the European Union member states, with reference to the objectives of Agenda 2030. The alternatives analysed were the 28 Member States belonging to the European Union, before Brexit (Fig. 2). They were compared by evaluating their progress according to the 2030 Agenda Sustainable Development Goals between 2007 and 2017, considering data corresponding to three years (2007; 2012;2017).

**Fig. 3:**
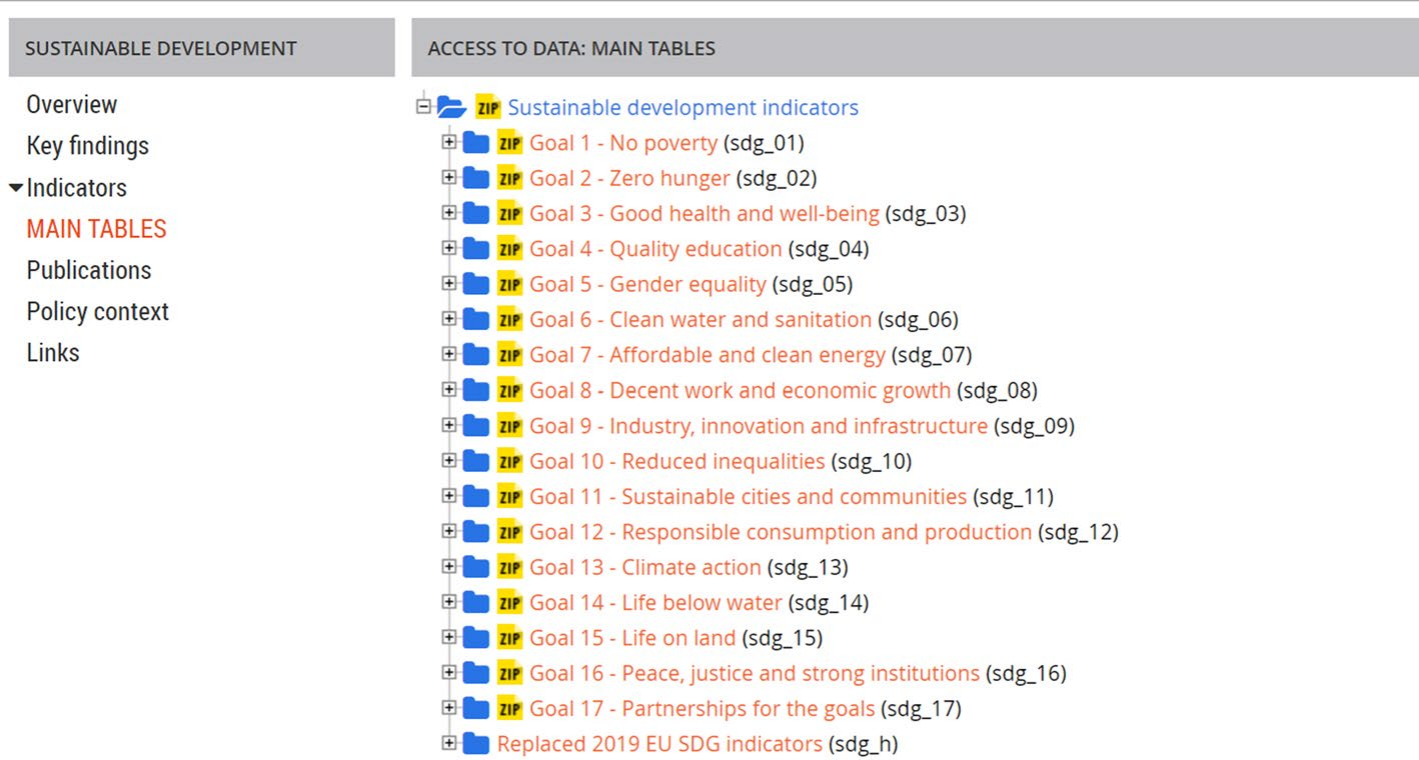
Sustainable development indicators database and list of indicators for Goal 13- Climate actions. Source: https://ec.europa.eu/eurostat/web/sdi/main-tables

The EU commitment is to implement the 2030 Agenda reinforcing sustainability policies amongst the states of the community. To trace the progress in reaching the SDGs through the formulation of sound policies, an indicators-based monitoring system has been built. For each goal, some indicators have been set for monitoring purposes in the Eurostat database (Fig. 3).

For assessing the results of the EU member states according to the Agenda 2030, 40 indicators have been selected, covering the 17 goals (Table 1). All the chosen indicators are regarded as “significant” according to the document “Sustainable development in the European Union” (Eurostat, 2017) and they are available in terms of data, for the time interval considered (years 2007–2012–2017). Anyway, similar studies can be carried out considering other indicators, if all the data are available.

**Table 1:** Goals and indicators considered in the analysis

Goals	Indicators	units
Goal 1 – No poverty	1.1 – People at risk of poverty or social exclusion	%
	1.2 – People at risk of income poverty after social transfers	%
Goal 2 – Zero hunger	2.1 – Agricultural factor income per annual work unit	€/AWU
	2.2 – Government support to agricultural research and development	€ per capita
Goal 3 – Good health and well-being	3.1 – Life expectancy at birth	n
	3.2 – Share of people with good or very good perceived health	%
	3.3 – Self-reported unmet need for medical examination and care	%
Goal 4 – Quality education	4.1 – Early leavers from education and training	%
	4.2 – Employment rates of recent graduates	%
	4.3 – Adult participation in learning	%
Goal 5 – Gender equality	5.1 – Gender pay gap in unadjusted form	%
	5.2 – Gender employment gap	%
	5.3 – Positions held by women in senior management positions	%
Goal 6 – Clean water and sanitation	6.1 – Population having neither a bath, nor a shower, nor indoor flushing toilet in their household owing to poverty status	%
Goal 7 – Affordable and clean energy	7.1 – Primary energy consumption	MTEP
	7.2 – Energy productivity	KGOE
	7.3 – Share of renewable energy in gross final energy consumption	%
Goal 8 – Decent work and economic growth	8.1 – Real GDP	€ per capita
	8.2 – Employment rate	%
	8.3 – People killed in accidents at work	per 100 000 persons in employment
Goal 9 – Industry, innovation and infrastructure	9.1 – Gross domestic expenditure on R&D by sector	%
	9.2 – Share of buses and trains in total passenger transport	%
Goal 10 – Reduced inequalities	10.1 – Purchasing power adjusted GDP	%
	10.2 – Relative median at-risk-of-poverty gap	%
Goal 11– Sustainable cities and communities	11.1 – Overcrowding rate owing to poverty status	%
	11.2 – Exposure to air pollution by particulate matter	µg/m3
	11.3 – Recycling rate of municipal waste	%
Goal 12 – Responsible consumption and production	12.1 – Circular material use rate	% of material input for domestic use
	12.2– Resource productivity and domestic material consumption	PPS (Purchasing power standard) per Kg
Goal 13 – Climate action	13.1 – Greenhouse gas emissions	Co2 Teq
	13.2 – Average CO2 emissions per km from new passenger cars	g Co2/Km
Goal 14 – Life below water	14.1 – Bathing sites with excellent water quality	%
	14.2 – Surface of marine sites designated under Natura 2000	Km2
Goal 15 – Life on land	15.1 – Surface of terrestrial sites designated under Natura 2000	%
	15.2 – Soil sealing index	%
Goal 16 – Peace, justice and strong institutions	16.1 – Population reporting occurrence of crime, violence or vandalism in their area owing to poverty status	%
	16.2 – Corruption Perceptions Index	0–100
	16.3 – Population with confidence in EU institutions	%
Goal 17 – Partnerships for the goals	17.1 – EU imports from developing countries	000 €
	17.2 – General government gross debt	%

**Fig. 4:**
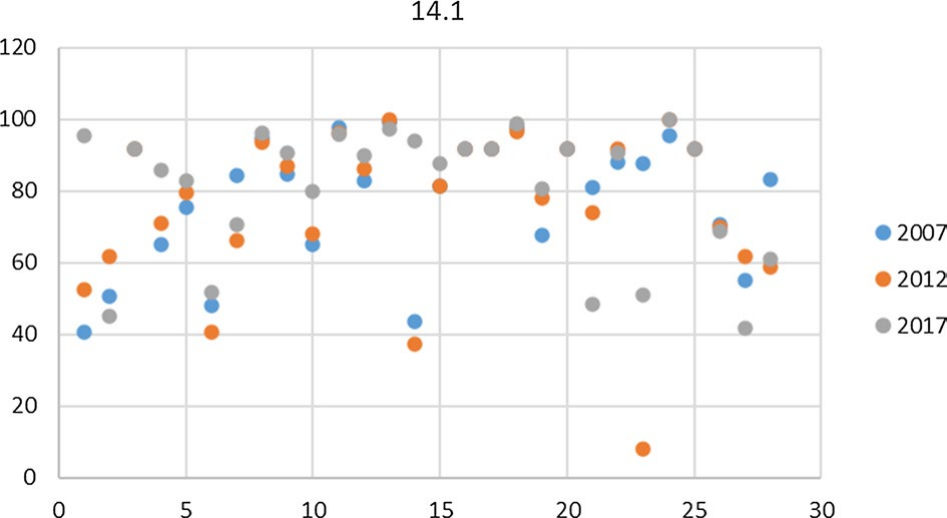
Scatter plot for indicator 14.1- Bathing sites with excellent water quality (% excellent coastal water)

For the calculation of the composite indicators, it is necessary to determine the reference levels ${q}_{i}$ which define the performance of each indicator *i* in relation to the Goal (*q*) in which it is included. In addition to the minimum (Min) and maximum (Max) values, three other intermediate levels corresponding to the percentiles 25 (P25), 50 (P50) and 75 (P75), across all the countries considered, have been statistically fixed. Note that different levels are used for each of the three years considered. Consequently, the results will show the relative position, in each year studied, of each country with respect to all the EU-28 countries. it is worth noting that if absolute levels were defined by experts for each indicator, containing thresholds or desirable levels, they could be used instead of the statistical levels. In order to evaluate the dispersion of data and identify any outliers, a scatter plot was created for each indicator and each year (Fig. 4). A single anomalous value, numerically distant from the rest of the collected data, has been found just for the Indicator 14.1- Bathing sites with excellent water quality (% excellent coastal water) for year 2012 in Romania (8.16%). Said value was replaced with the nearest worst value, which is the one of Latvia (37.50%) for the same year, in order not to prejudice the final result of the analysis.

**Fig. 5:**
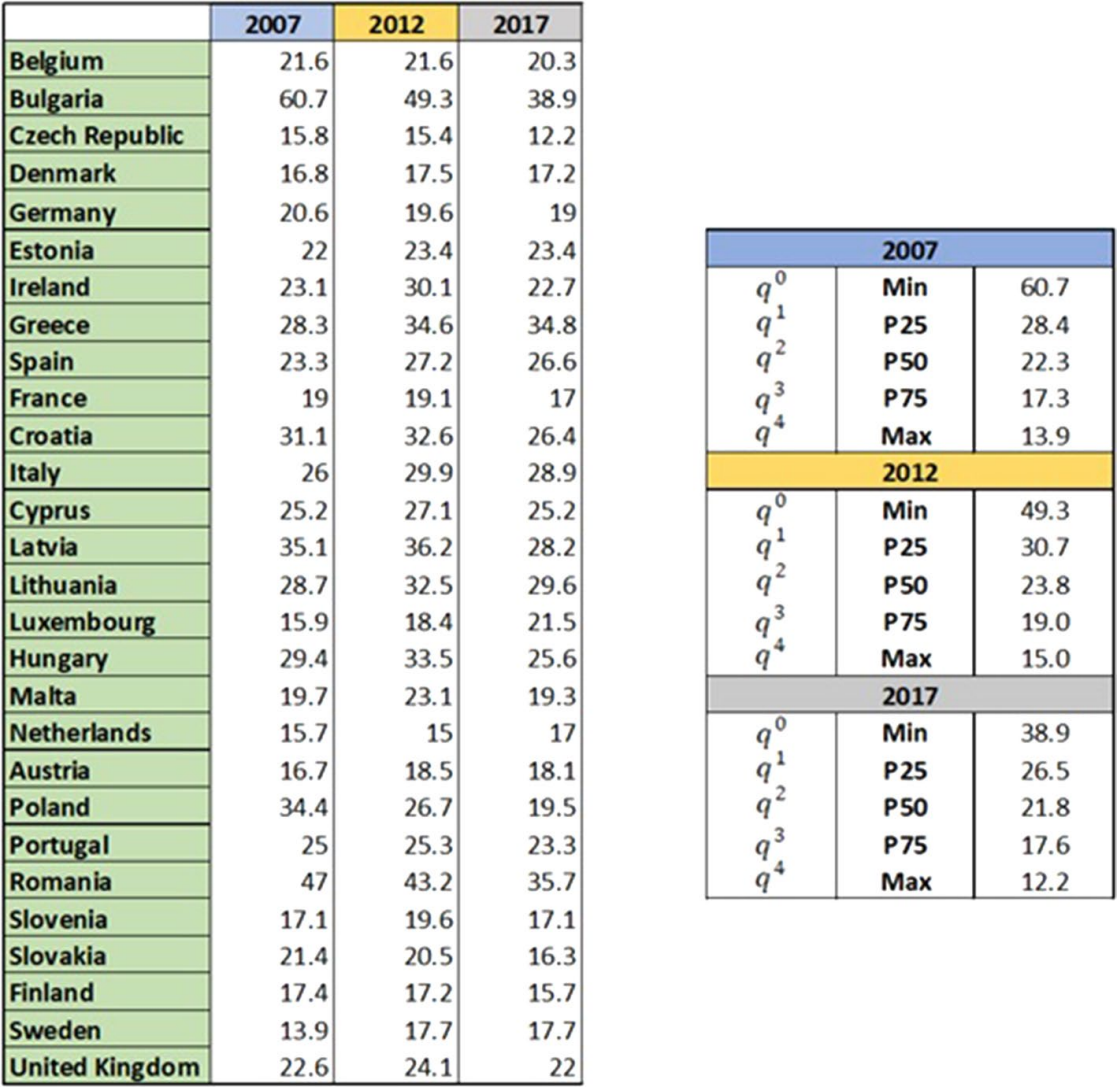
Example for indicator 1.1—People at risk of poverty or social exclusion: data for each year and reference levels

Figure 5 reports an example for indicator 1.1 of all the information needed in the analysis: the data for each country and the statistical values identified as minimum, maximum and percentiles (P25-P50-P75) for each year. As previously mentioned, no prior normalisation of the indicators is needed, given that the achievement function brings all the indicators down to a common 0-1-2-3-4 scale. The sustainability levels associated with the different values obtained for an achievement function *s* are displayed in Table 2.

**Table 2:** Achievement function and levels of sustainability

Interval	Level of sustainability
$0\le s\le 1$	Insufficient
$1<s\le 2$	Sufficient
$2<s\le 3$	Good
$3<s\le 4$	Excellent

**Fig. 6:**
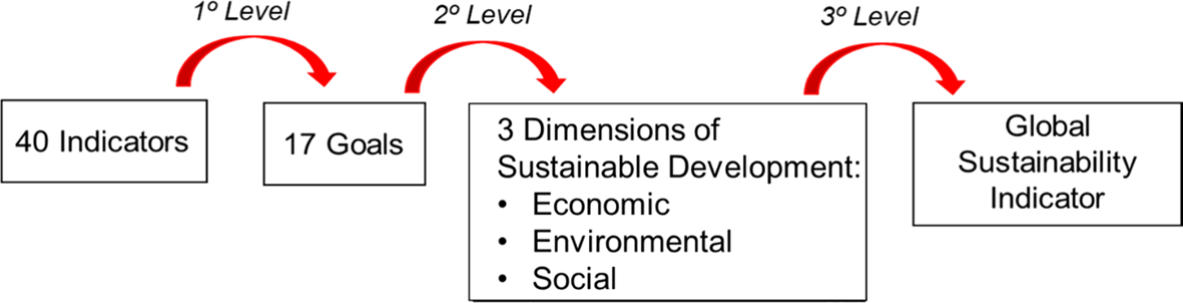
Aggregation steps for each EU Member and for each year considered (2007–2012-2017)

Indicators have been aggregated in three levels. The first aggregation level is amongst indicators, to obtain one index for each sustainable development goal. Then, each of the 17 SDGs has been classified under one of the three sustainability dimensions (Economic, Environmental, Social) and the aggregation has been carried out within each dimension. Finally, the three sustainability dimensions have been aggregated in a global sustainability index. The three aggregations have been repeated for the three reference years (2007, 2012, 2017) for each EU member (Fig. 6), applying both the MRP-WSCI and MRP-PCI schemes.

The weighting phase is aimed at assessing the contribution of each indicator and goal to the final global value; the value 1 was chosen as the reference weight and according to this, double and triple have been used, that is weights equal to 2 and 3 (Table 3). For this weight’s allocation, three EU documents were considered:

- Europe 2020: A strategy for smart, sustainable and inclusive growth.^1^
- A Union that strives for more” Ursula Von Der Leyen electoral agenda.^2^
- Budget proposal for the 2021–2027 and new cohesion policy (May 2018).^3^

**Table 3:** weights scale

Assessment	Value
Important	1
Very important	2
Critically important	3

The first document is a strategic plan proposed by the European Commission in 2010: it has been the main road for European investment until 2020. The second document was presented by the European Commission in 2018 and illustrates the proposals of the new budget and regulations related to cohesion policy 2021–2027. Finally, the last one refers to the electoral and programmatic structure presented by the President of the European Commission. Although the three documents are different from each other, they share common traits, regarding the importance given to certain sectors. Undoubtedly, the central theme of the policies is to move towards a greener, low carbon emissions Europe. Hence, higher weights were assigned to Goal 7—Clean and affordable energy, Goal 11—Sustainable cities and communities and Goal 13—Climate action. With regard to the social sphere, in all the documents great attention has been paid to the issues linked to poverty (to be reduced by at least 25%), to a high school drop-out rate and gender inequality, especially in terms of the quality of work. This is why the highest weights were assigned to Goal 1—Overcoming poverty, Goal 4—Quality education and Goal 5—Gender equality. Finally, concerning the economic dimension, the central point is to increase investment in research and development in order to be able to go in an innovative direction suitable for the digital age. Therefore, the goal with weight 3 is Goal 9—Enterprise, innovation and infrastructure which contains the indicator 9.1 Gross domestic expenditure on R&D (% of GDP).

**Fig. 7:**
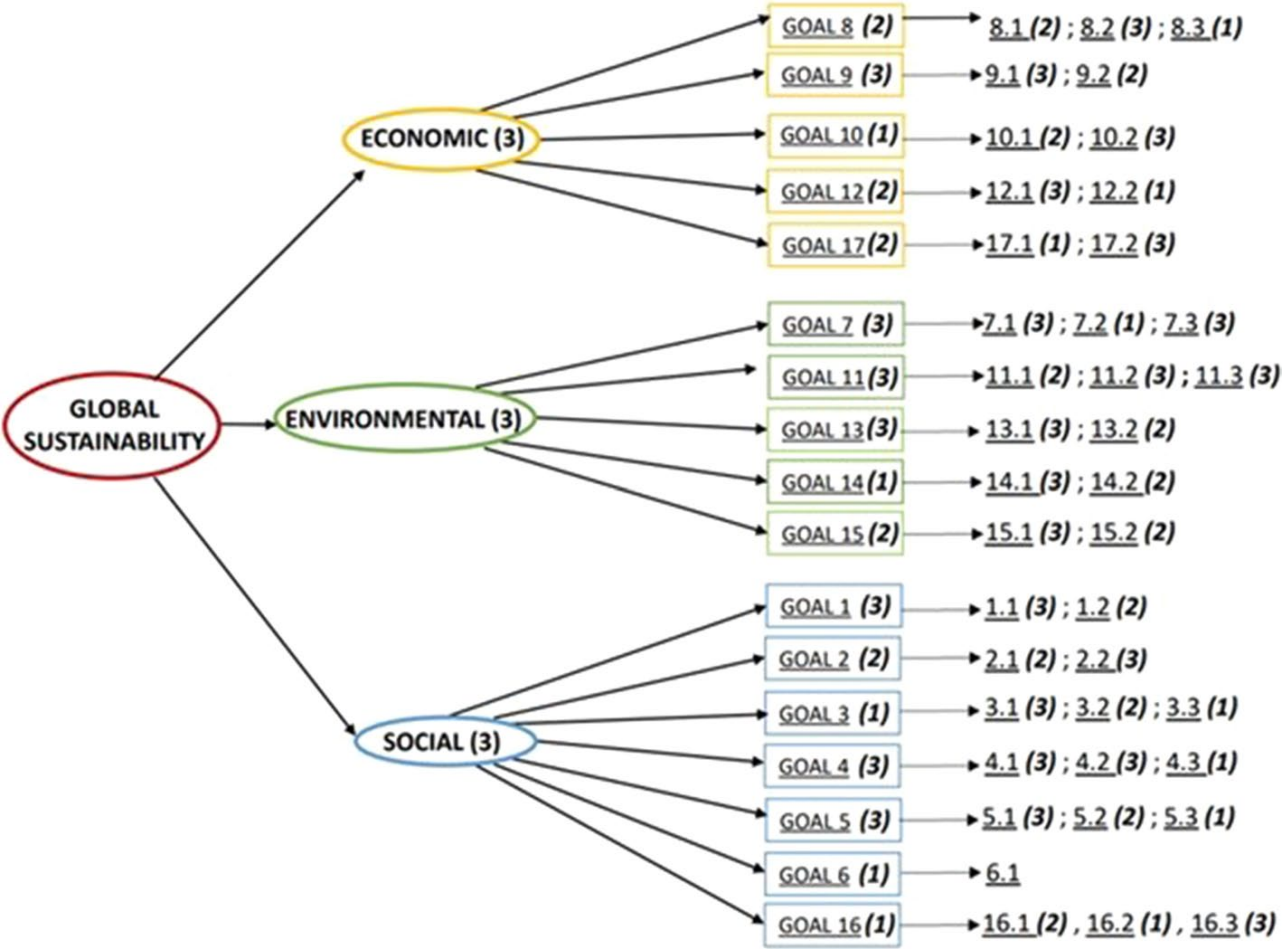
Weights used in the analysis and aggregation scheme

The results of the weighting phase and all the weights used in the analysis are reported in Fig. 7, along with the aggregation scheme.

The same aggregation scheme and weights have been used for applying the MRP-WSCI scheme, producing both a Weak-Weak-Weak (W-W-W) and Strong-Weak-Weak (S-W-W) global composite indicators, and for the MRP-PCI scheme, calculating a global Partially Compensatory Composite Indicator.

## Results and Discussion

### Weak-Weak-Weak (W-W-W) and Strong–Weak-Weak (S-W-W) Global Composite Indicators

The Weak-Weak-Weak Global Composite Indicator (W-W-W) and the Strong-Weak-Weak (S-W-W) are the global composite indices deriving from the third and last aggregation step of the application of MRP-WSCI. In the W-W-W, the Weak Composite Indicator (WCI) has been applied at each aggregation step, whilst in the S-W-W the Strong Composite Indicator (SCI) was applied in the final step. Therefore, the W-W-W represents a measure of global compensation: it provides a value that is the result of the average between the three dimensions of sustainable development, namely economic, social, and environmental ones. The S-W-W, on the other hand, allows the identification of the worst sustainability dimension. Running both indices is very advantageous, as it provides an image of global sustainability, but it also allows a specific dimension to be accessed. Moreover, the W-W-W very often turns in a good performance that depends on just two dimensions, while the third dimension has poor levels.

**Fig. 8:**
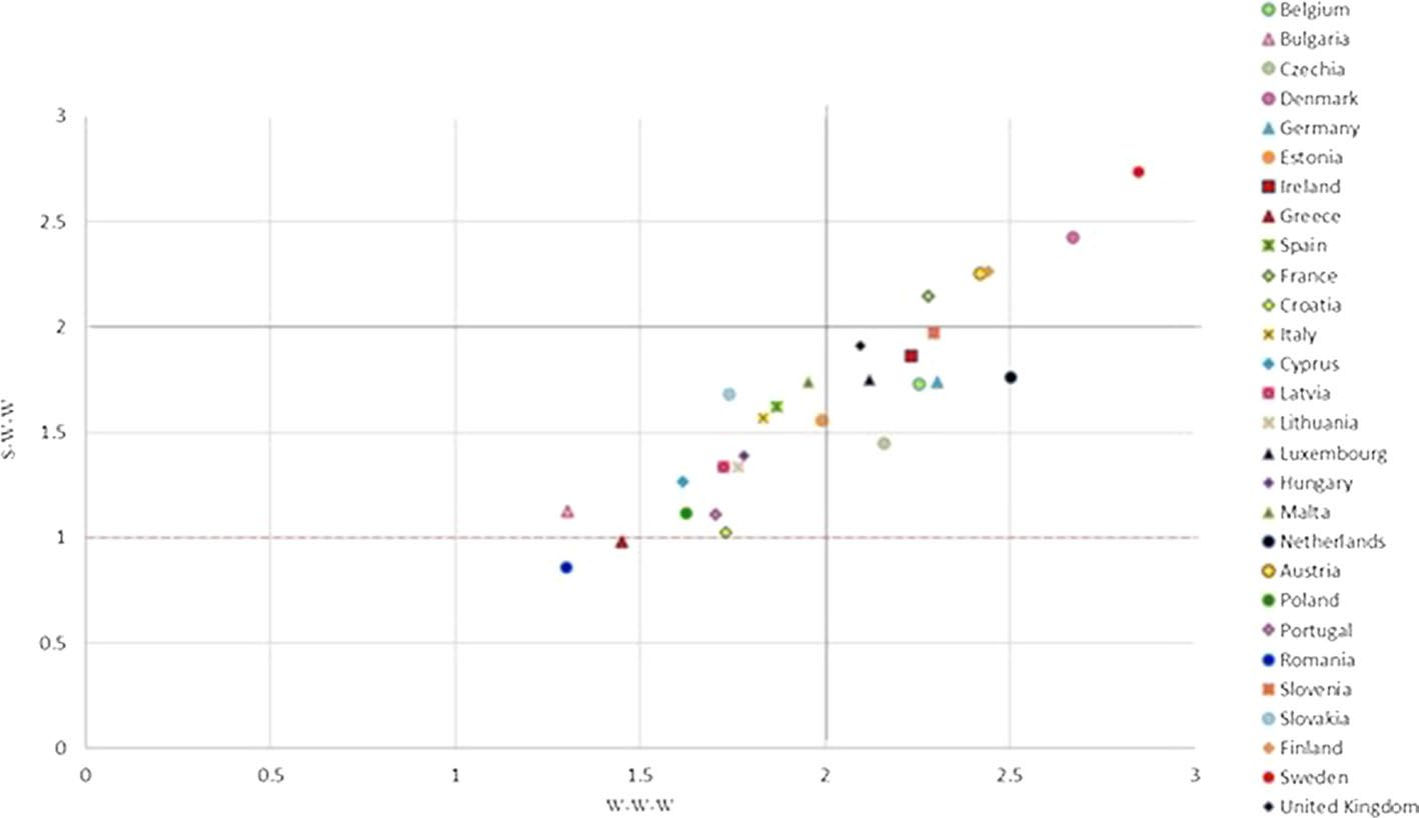
EU member states joint results 2017

Figure 8 reports at the same time the results of the W-W-W (x axis) and of the S-W-W (y axis) for the different EU countries. As previously mentioned, considering the common scale, based on the reference levels, between 1 and 4, the values will be considered as follows:

- Over percentile 75 if they are greater than 3;
- Between percentiles 50 and 75, if they are between 2 and 3;
- Between percentiles 25 and 50, if they are between 1 and 2;
- Under percentile 25, if they are less than 1.

Given this classification, the EU countries can be classified as follows, according to the sustainability level:

- Excellent, if both W-W-W and S-W-W are between percentiles 50 and 75;
- Good, if only W-W-W is between percentiles 50 and 75;
- Sufficient, if both W-W-W and S-W-W are between percentiles 25 and 50, but W-W-W is over 1.5;
- Insufficient, if only W-W-W is between percentiles 25 and 50, while S-W-W is under percentile 25.

Figure 8 is divided into four quadrants by value 2, while the red line at value 1 indicates the states that are below the sufficiency for the value of S-W-W. It gives a general and immediate idea of which countries have a good overall result and those which have the worst results. A more detailed one follows in which the EU states are analysed according to their positioning on the chart.

#### First Quadrant: States with an Excellent Level of Sustainability

**Fig. 9:**
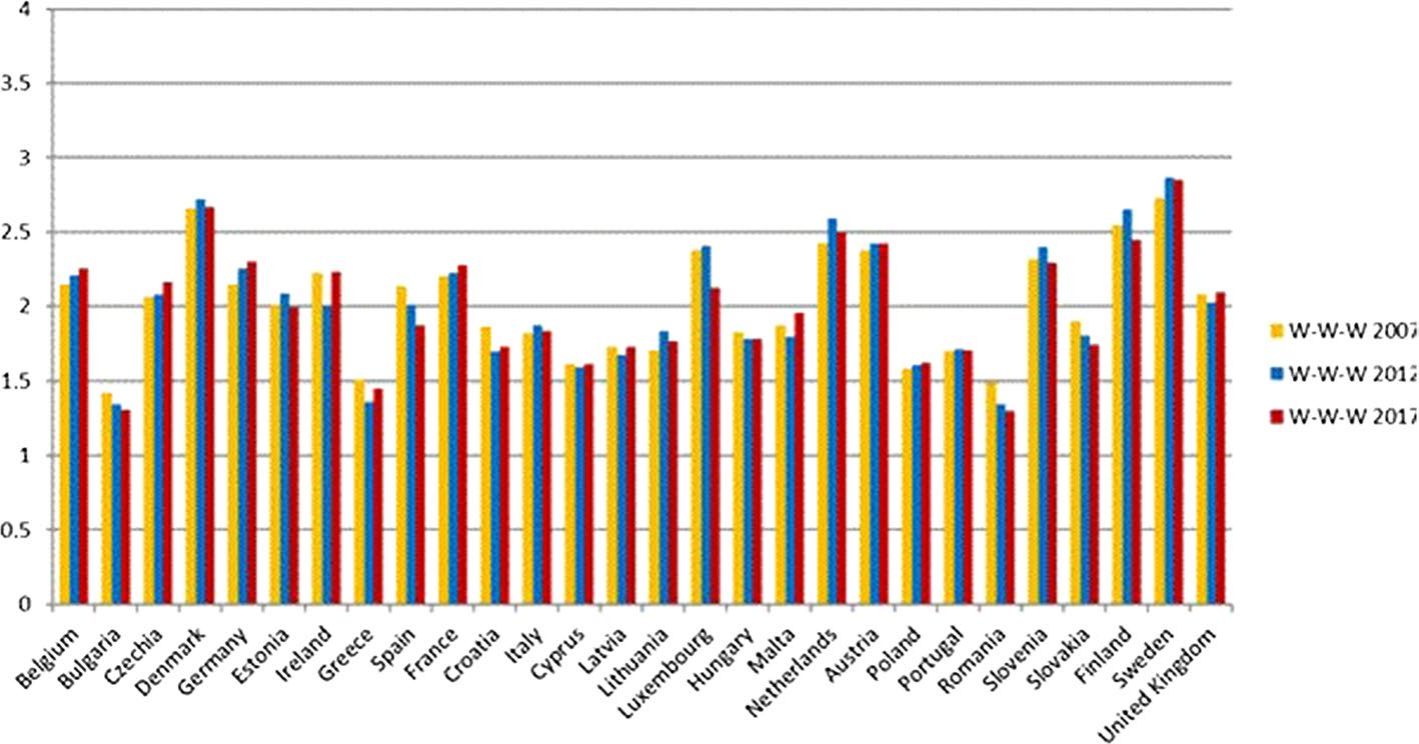
Global sustainability index W-W-W 2007, 2012, 2017

Sweden, Denmark, Finland, Austria and France are in the first quadrant and therefore they have excellent sustainability performances, considering both the global W-W-W and the S-W-W indicators. Furthermore, considering the dynamic evolution, Sweden, Austria, and France have also improved or maintained the value of the global W-W-W indicator in the 2007–2017 decade (Fig. 9), whilst the same does not apply for Finland and Denmark.

**Fig. 10:**
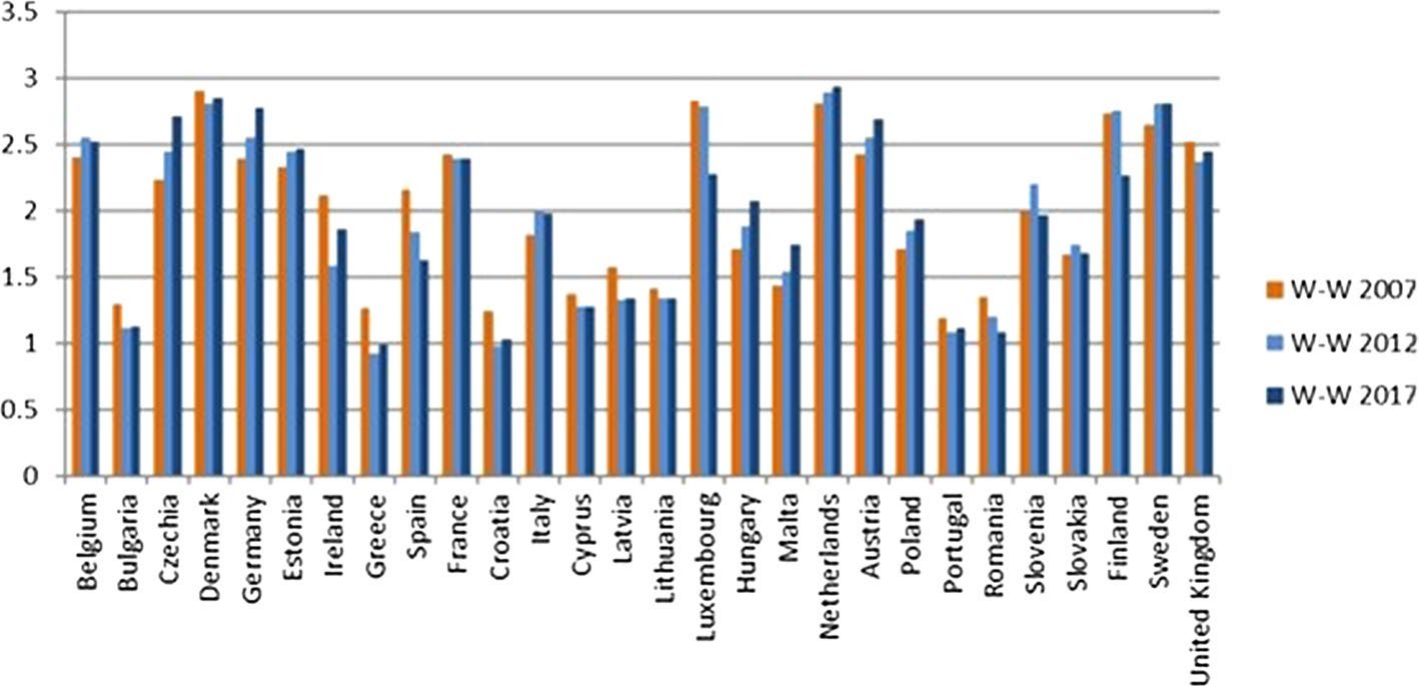
Economic Sustainability index W-W 2007, 2012, 2017

**Fig. 11:**
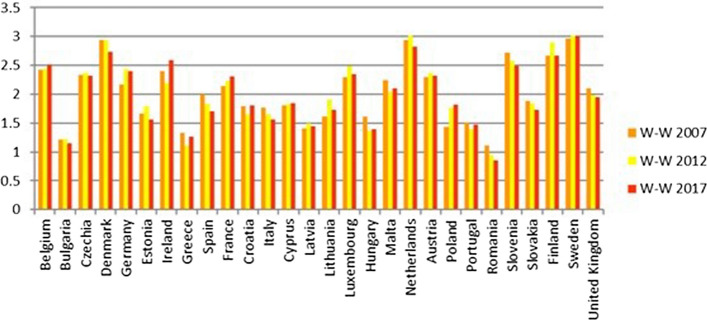
Social Sustainability index W-W 2007, 2012, 2017

Finland has got worse mainly due to the reduction in the values of the economic dimension between 2012 and 2017 (Fig. 10). In fact, the values of indicators 8-9-10–12-17, belonging to the economic dimension, have decreased. On the contrary, for Denmark the decrease is mainly due to the social dimension (Fig. 11), and in particular the indicators of SDG 4 “Quality education” have suffered a strong decrease.

#### Second Quadrant: States with a Good Level of Sustainability

**Fig. 12:**
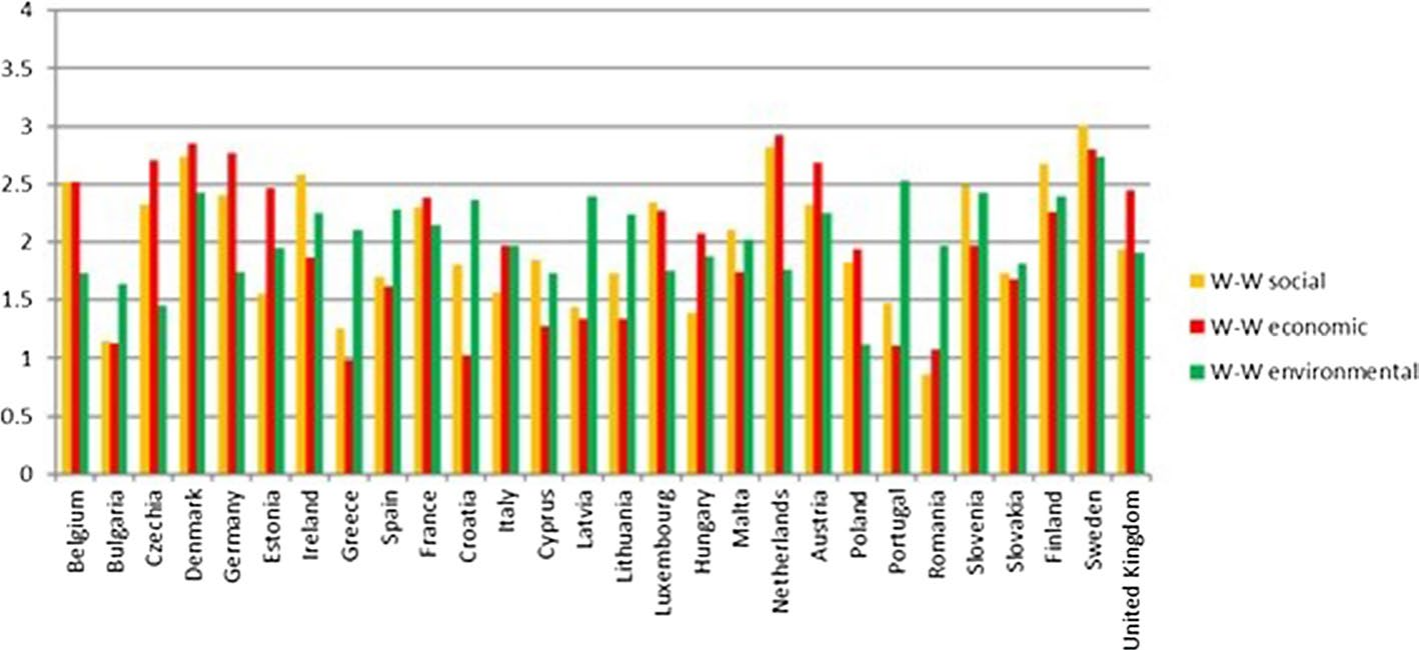
Composite sustainability indices for the three dimensions, 2017

The Netherlands, Ireland, Slovenia, Germany, Belgium, United Kingdom, Luxembourg and the Czech Republic are in the second quadrant; they are thus classified as good (Fig. 8). The value of the global indicator W-W-W is greater than 2 and they are also sufficient according to the S-W-W. However, values of S-W-W below 2 highlight difficulties at the level of one or more dimensions. For instance, Fig. 8 shows that the Netherlands have a better global index than Austria and Finland, but certainly the value of one of the dimensions is not as good. This is also confirmed by Fig. 12 (W-W Composite sustainability indicator for the three sustainability dimensions), which shows, for year 2017, a more than good value for the economic and social dimensions (both over 2.5), whilst the environmental one is below 2.

**Fig. 13:**
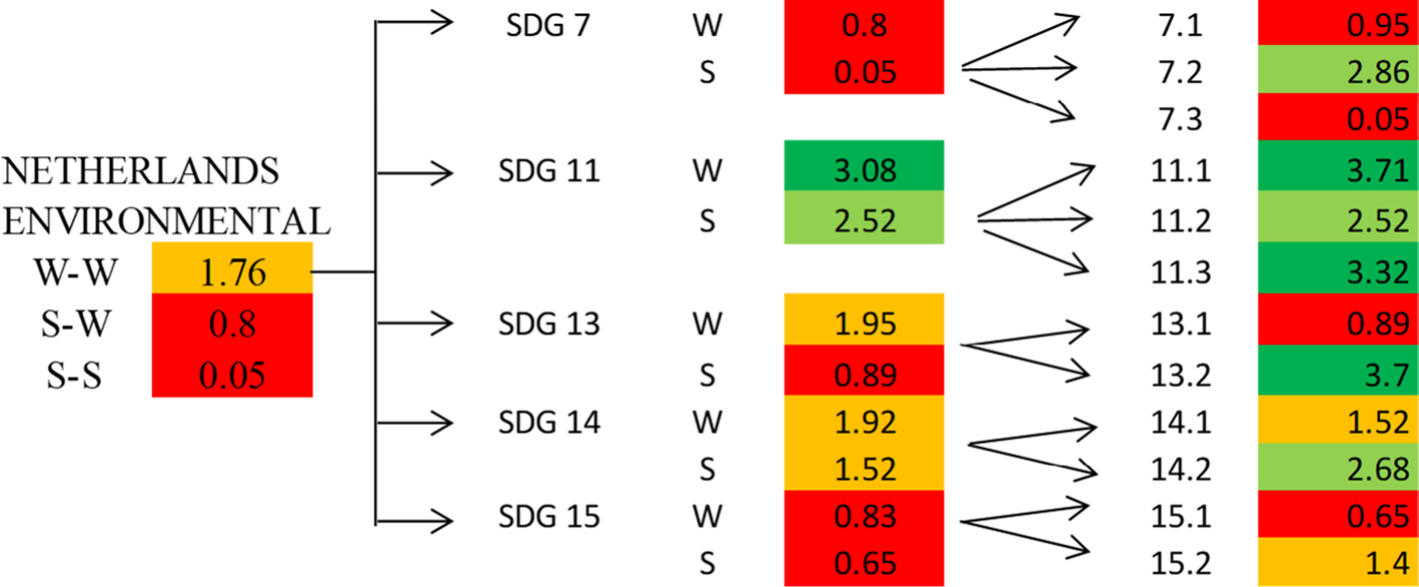
Composite sustainability indices for the three dimensions, 2017

Going into a more in-depth analysis for the Netherlands, Fig. 13 highlights that the bad result for the environmental dimension is mainly due to the results of SDG 7 “Affordable and clean energy” and SDG 15 “Life on land”. Such an output is a confirmation of what Delli and Paoli found out about some countries, such as the Netherlands, which are amongst the worst performers in terms of land sustainability (SDG 15) and against climate change (SDG 13). The same conduct is shown also by Czech Republic, Luxembourg, Germany and Belgium (Fig. 12).

In 2017 Luxembourg registered a considerable drop in the value of the W-W-W index (Fig. 9), although only the value of the environmental dimension is not good (Fig. 12). However, the decrease over the years has to be considered carefully, as the fall in values involved both the economic (Fig. 10) and environmental (Fig. 12) dimensions.

**Fig. 14:**
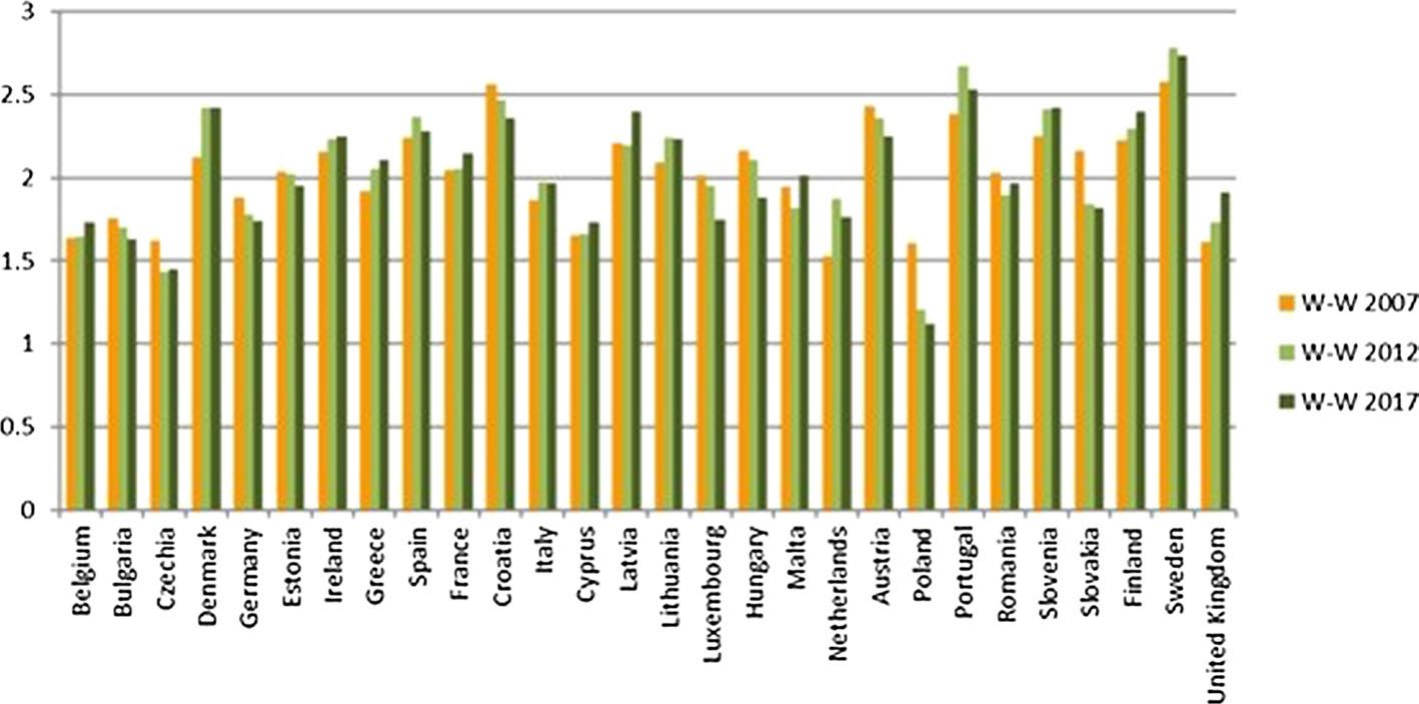
Environmental Sustainability index W-W 2007, 2012, 2017

Germany has a value of the environmental indicator W-W equal to 1.73 in 2017 and always under 2 for the period considered (Fig. 14). However, all its objectives reach sufficient values, except for GOAL 13—Climate action, which has both indicators involved at a level less than 1.

Finally, Ireland and Slovenia are in this group because they have values below 2 for the economic dimension. Only United Kingdom has values below 2 for two dimensions, the social and environmental ones (Fig. 12).

#### Third Quadrant: States with a Sufficient Level of Sustainability

Estonia, Malta, Italy, Spain, Slovakia, Hungary, Latvia, Lithuania, Cyprus, Poland, Portugal, Bulgaria, Croatia, Greece are located on the third quadrant and have a low value for both global composite indicators W-W-W and S-W-W. It can thus be deduced that these states have a bad value, i.e. below 2, for more than one dimension.

Estonia, Malta, Italy, Spain, and Slovakia have no dimensions less than 1.5 (Fig. 12), therefore these last four are at least sufficient. The weakest dimension for Estonia is the social one (Fig. 12), which has a very low value compared to the other two dimensions. Taking a closer look at the social dimension, only two objectives have good values, while all the others are just sufficient, and one is insufficient.

Malta has improved over the past decade, as noted in Fig. 8, while Spain and Slovakia have worsened, and Italy has not seen any major changes.

Hungary, Latvia, Lithuania, Cyprus, Poland, Portugal and Croatia have W-W-W values greater than 1.5, while S-W-W values are between 1 and 1.5. As for Croatia, Cyprus, Lithuania, Latvia and Portugal, the lowest value is reached by the economic W-W (Fig. 12), while Hungary registers the worst results in the social dimension and Poland in the environmental one. It is interesting to note that, although it is in the group of the worst countries, Portugal achieves the second-best value for the environmental dimension; moreover, it is the only country, along with Sweden, to obtain a result greater than 2.5 in this dimension.

#### States with an Insufficient Level of Sustainability

Bulgaria, Greece and Romania are the countries with the worst sustainability performance. They are in the third quadrant and they register the lowest values for both indicators (Fig. 8). The same results are shown in Delli Paoli et al. (2019): the countries with the worst results of the global sustainability indicator are the same. On a scale between 0 and 100, in which 0 is the worst result and 100 the best, Greece has a value of 7, Bulgaria 1 and Romania is completely unsuccessful with a value of 0. Additionally, these negative results are mainly due to the economic and social dimensions.

In particular, Romania and Greece have one or more dimensions below the sufficiency threshold considered. According to Fig. 12, the problem is mainly at economic and social levels, while Greece reaches good levels for the environmental dimension and the other two states show an environmental W-W above 1.5. In Firoiu et al. (2019), the Romania’s results in terms of the SDGs were analysed considering the 2017 results. It was found that the indicator values of the economic and social dimensions are very distant from the EU average, while those of the environmental dimension are generally sufficient, except for SDG 11 (Goal 11– Sustainable cities and communities).

The values found in the social indicators were compared to those analysed by Ionescu et al. (2020). They included the SDGs 1–3-4-5–10–16 in the social sphere, while in our work we did not consider goal 10, but goals 2 and 6. The results are very similar; in fact, it can be noticed that the countries of the first quadrant almost always register positive values, whilst those of the last quadrant generally have values much lower than those of the European average. In addition, we found the same results by analysing the trend for 2030; the best countries showed a positive trend, while the worst states showed a negative trend in achieving the sustainable development goals.

The social and economic pillars are quite aligned, whereas the environmental one is disconnected from the other two. As a matter of fact, the states with the worst results in global sustainability get their best results for the environmental dimension. This happens for the countries of the third quadrant such as Slovakia, Spain, Latvia, Lithuania and Portugal and as Bulgaria, Romania and Greece. On the other hand, we see opposite outcomes from countries characterised by a good level of global sustainability and a high value in social and economic dimensions.

By considering Fig. 12, it is clear that some of the countries that are better ranked have their lowest value for the environmental dimension. This is particularly true of Denmark, France and Sweden (first quadrant) and Belgium, Czech Republic, Germany, Luxembourg, Netherlands and the United Kingdom (second quadrant). In terms of absolute values, Sweden and Denmark are still at the top of the list (first and third), but the difference with the worst countries is much smaller in the environmental sphere than in the economic and social ones. Belgium, Czech Republic, Germany, Luxemburg, Netherlands and United Kingdom are actually at the bottom of the ranking, with values that do not reach very good levels of sustainability, in actual fact, less than two.

### Partially Compensatory Composite Indicator

The Partially Compensatory Composite Indicators obtained in each aggregation phase were used as achievement functions to determine the composite indicator of the next step. In particular, for the PCI, in each step a compensation index ${\lambda }_{i}$ was provided. ${\lambda }_{i}$ ranges between 0 and 1, according to the degree of compensation:

- First aggregation: ${\lambda }_{i}=1$, perfect compensation among indicators belonging to the same goal.
- Second aggregation: ${\lambda }_{i}=0.5$, partial (medium) compensation among goals belonging to the same dimension.
- Third aggregation: ${\lambda }_{i}=0$, according to strong sustainability, no compensation is allowed among the three dimensions.

Amongst indicators in the same Goal, total compensation (λ_*i*_ = 1) has been allowed. The assumption is that the indicators with bad values can be compensated by the ones achieving better performance if they belong to the same Goal.

Moving away from the field of individual indicators through aggregations, it is assumed that the decision maker will implement less and less compensation between composite indicators obtained according to the theory of strong sustainability. Contrary to weak sustainability, strong sustainability assumes that "human capital" and "natural capital" are complementary, but not interchangeable. Thus, partial (medium) compensation (λ^*i*^ = 0.5) is considered for the goals belonging to the same dimension, whilst in the last aggregation, amongst the dimensions of sustainable development, λ_*i*_ = 0 (no compensation) has been used. The result obtained reflects the worst value achieved in one of the three dimensions of sustainability, but considering the partial compensation used at the second aggregation and according to the principles of strong sustainability, the three dimensions have the same weight.

This choice has been taken because strong sustainability supports that certain functions performed by the environment cannot be duplicated by human beings or human capital and it emphasises the ecological scale with respect to economic gains. This implies that nature has the right to exist and that it has been borrowed and should be passed on from one generation to the next one intact in its original form.

As the common scale goes from 0 to 4, if a member state is in the range from 0 to 1, it presents an unsustainable situation; within the interval between 1 and 2, countries are on the threshold of sustainability sufficiency; between 2 and 3 there is a good level of sustainability and, finally, if the value is greater than 3 the state has an optimal level of sustainability.

**Fig. 15:**
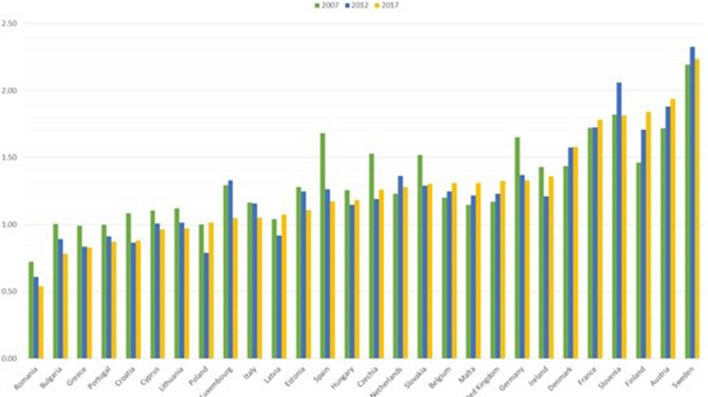
Global PCI over years

Analysing the decade considered (Fig. 15), most of the States resulted in a global PCI between 1 and 2 with many fluctuating trends. There is thus a sufficient level of sustainability amongst countries, although the situation is still far from an optimum one. A value greater than 2 was achieved only by Sweden in 2017 (2.24), while above 1.5, we can find Austria, Finland, Slovenia, France, and Denmark. Belgium, Malta, the United Kingdom, Finland, and Austria have progressively improved over the decade. By contrast, Germany and Spain showed a significant and progressive deterioration. Lithuania, Cyprus, Croatia, Portugal, Greece, Bulgaria, and Romania closed the considered period with a value below 1 in 2017. It should be noted that those last two countries achieved the worst values, which are 0.78 and 0.54, respectively.

As the global PCI of each State reflects the value of the worst dimension among the three, it is possible to divide the countries into groups. For Belgium, Czech Republic, Denmark, Germany, Estonia, France, Luxembourg, Malta, Holland, Poland, Sweden, and United Kingdom, the worst dimension is the environmental one. Instead for Hungary, Spain, Italy, Hungary, Austria, Romania and Slovakia, the worst dimension is the social one. Finally, for Ireland, Greece, Croatia, Cyprus, Latvia, Lithuania, Portugal, Slovenia and Finland, the worst dimension is the economic one (see Table 4).

**Table 4:** Worst dimension in PCI, global and pillars values

2017	Global	Social	Economic	Environmental
Belgium	1.31	2.23	2.06	**1.31**
Bulgaria	0.78	**0.78**	0.95	1.20
Czechia	1.26	1.49	2.56	**1.26**
Denmark	1.58	2.34	2.63	**1.58**
Germany	1.33	2.05	2.70	**1.33**
Estonia	1.11	1.23	2.20	**1.11**
Ireland	1.36	2.20	**1.36**	2.01
Greece	0.83	0.88	**0.83**	1.44
Spain	1.17	**1.17**	1.31	1.93
France	1.78	2.00	2.03	**1.78**
Croatia	0.88	1.32	**0.88**	1.42
Italy	1.05	**1.05**	1.73	1.83
Cyprus	0.96	1.66	**0.96**	1.49
Latvia	1.07	1.07	**1.07**	1.95
Lithuania	0.97	1.29	**0.97**	1.99
Luxembourg	1.05	1.66	2.09	**1.05**
Hungary	1.18	**1.18**	1.90	1.50
Malta	1.31	1.38	1.35	**1.31**
Netherlands	1.28	2.35	2.52	**1.28**
Austria	1.94	**1.94**	2.21	2.04
Poland	1.01	1.26	1.65	**1.01**
Portugal	0.87	1.18	**0.87**	2.25
Romania	0.54	**0.54**	0.74	1.23
Slovenia	1.81	2.01	**1.81**	2.27
Slovakia	1.30	**1.30**	1.62	1.47
Finland	1.84	2.32	**1.84**	2.05
Sweden	2.24	2.83	2.35	**2.24**
United Kingdom	1.32	1.43	2.12	**1.32**

Bold values indicate the worst dimension (value) for each country

### MRP-WSCI and MRP-PCI: A Comparison of Results

**Fig. 16:**
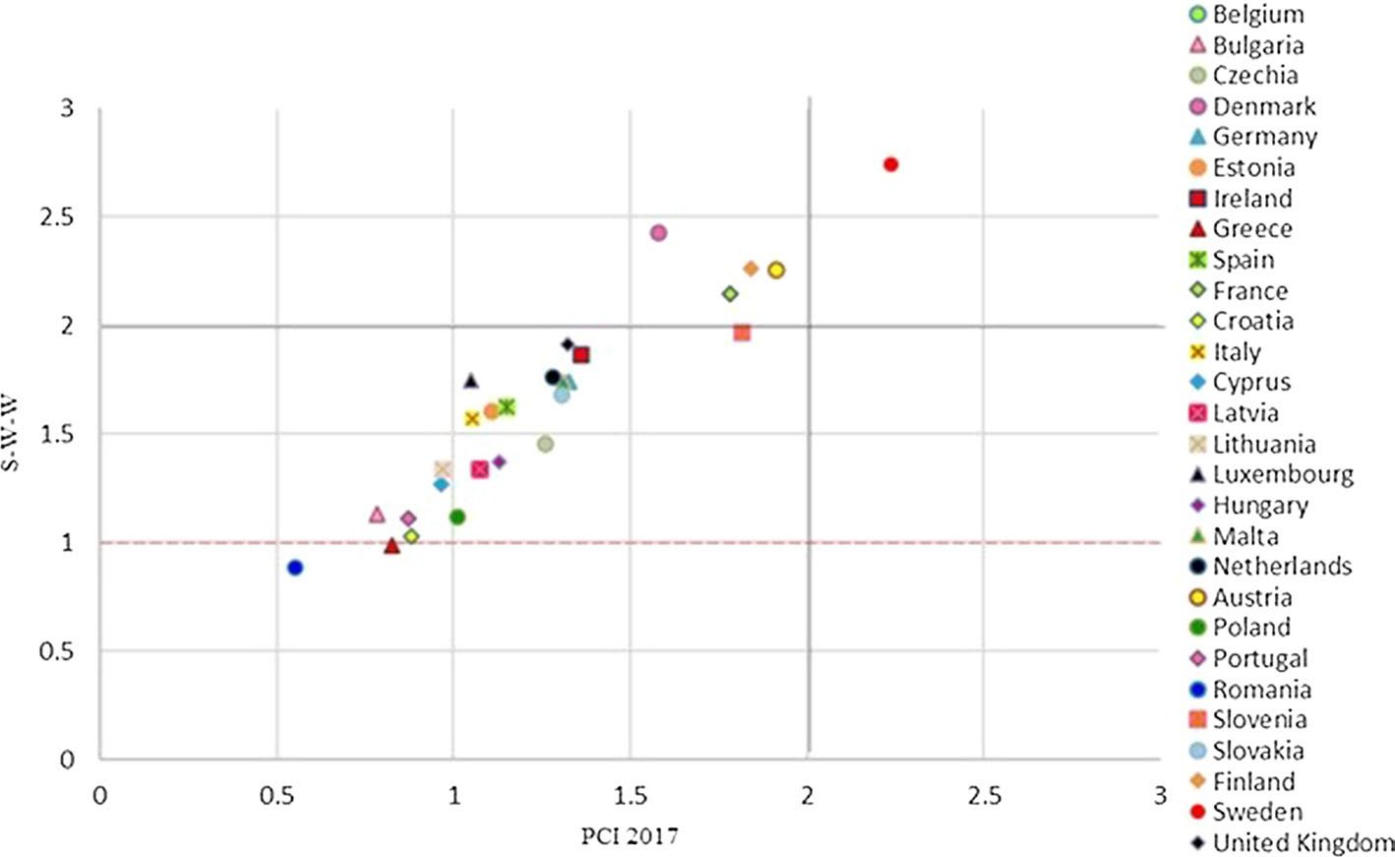
Scatter plot between S-W-W and PCI for 2017

The MRP-PCI method permits the Decision Maker to assign different compensation indices to each indicator (or each family, subfamily, etc.) of the system. This allows the consideration of different indicators as differently compensable, instead of providing a single compensation index for the entire system as previously done in the MRP-WSCI scheme. For year 2017, a comparison between the last aggregation of S-W-W and PCI has been carried out (Fig. 16). Given the compensation indices used for the MRP-PCI scheme, the differences between the solutions obtained with both approaches is due to the partial compensation used in the second aggregation for the PCI indicator.

The PCI registered a fall of between − 0.1 and 0.84 points in comparison to the S-W-W, for each country.

The country with the highest deviation between the two values is Denmark, which has PCI = 1.58, less than S-W-W = 2.42; this difference is due to the distinct degree of compensation used in the 3 aggregations. In the first one, full compensation was allowed in both cases; thus, the value of the weak composite indicator and the PCI are equal. In the second level of aggregation, we consider the value of the composite indicator W-W, as it is used as a scalarizing function for the construction of the composite indicators of the next phase. The value of the PCI is lower than W-W, especially with regard to the environmental dimension, where there are greater differences (PCI = 1,58, W-W = 2,42). This is because in the W-W there is a total compensation and, therefore, some Goals with bad values, such as the Goal 15 that has a value of 0.6 (insufficient), are compensated by those with good rate. On the other hand, in PCI there is partial compensation, so the worst Goals are not fully compensated, and these are reflected in the final value. The difference in global values of PCI and S-W-W is due to this last aggregation, because in the third level for both cases there has been no compensation; indeed, the values, although different, coincide with the worst sphere, the environmental one.

### Robustness Analysis

A robustness analysis has been carried out in order to measure the method’s reliability, evaluating the capacity of the methodology to remain unaffected by small, deliberate variations in method parameters, and the stability of the results obtained.

In particular, the robustness analysis has been carried out on the weights. The assigned weights have been randomly varied, ranging from ± 10% and ± 20%, using 100 repetitions. The analysis was only carried out for the CI of 2017, because if the weights are reliable in one year, it is sensible to consider them as such for the previous years too, since they have been assigned following the same methodology.

**Fig. 17:**
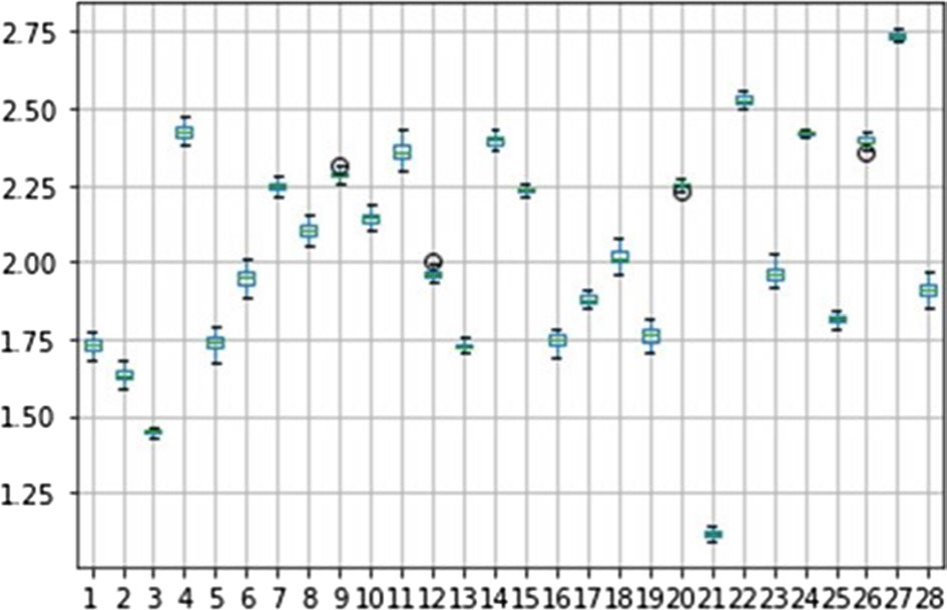
Robustness analysis of environmental dimension (W-W indicator)

Figure 17 presents the analysis for the environmental dimension (W-W) with weights varying by ± 10%. As can be noticed, despite a few outliers (circled in the figure), the variation ranges of the results showed in the box plot are fairly small. The figure thus shows that the results remain fairly stable as the weights vary. This confirms that the methodology used for this study is reliable, given the consistency of the results.

**Fig. 18:**
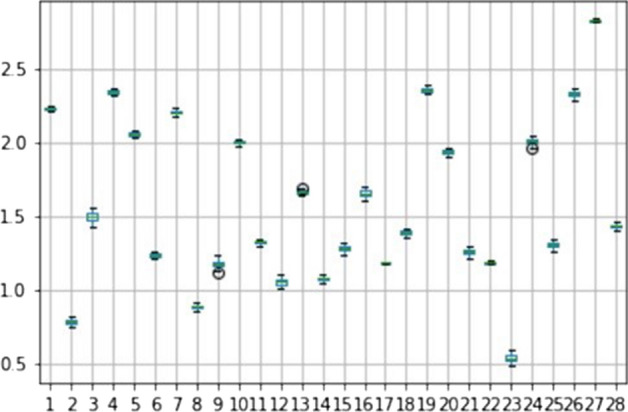
Robustness analysis of social dimension (PCI indicator)

The same procedure was used for the PCI indicator. Figure 18 shows the results of the robustness analysis for the social dimension, considering a variation by ± 20%. Even in this case, the values do not present significant variations and these results give us confirmation of the accuracy of the analysis.

## Discussion

One of the top priorities related with sustainability is to be able to identify and measure the level through its three dimensions. This process requires adequate technical support. In general, the basis for good decision-making is founded on ex ante evaluation, the monitoring of progress and ex-post evaluation.

Amongst the various Multi-criteria methods, composite indicators seem particularly capable of capturing and condensing all the necessary information.

The study includes the temporal dimension, considering three different years (2007–2012–2017). This allows us to include a period of 10 years and to analyse the progress or exacerbation of the performance of each State, thanks to the assessment of the individual countries through the data for the decade. Moreover, monitoring the developments of each individual country over the years is important to address further actions to correct any poor performances.

Sustainable development and SDGs show that it is not enough to calculate the indices and rankings of countries through separate points over time. Instead, in order to effectively monitor progress towards the harmonisation of Agenda 2030, it is also necessary to consider development over time. (Hametner & Kostetckaia, 2020). Even according to Miola and Schiltz (2019), the ranking of countries is not an appropriate approach to Agenda 2030, since the search for the best results is not its aim. Actually, in some countries, the process of implementing the SDG framework could be more important than the final performance outcome.

Furthermore, in their study they stress that differences in political priorities between countries could exaggerate the importance of choosing one or more methods or indicators. For example, countries that deliberately progress faster in specific indicators at the expense of others will be at a disadvantage when each indicator is afforded the same weight. From their perspective, it would be interesting to focus exclusively on countries that explicitly claim to place equal weights on sub-components of each SDG, and vice versa.

To overcome this issue, the present paper included the political dimension, through the use of weights both at the level of indicators and Goals, deriving from European strategic documents. In addition, the method makes it possible to have both an assessment for the three separate pillars and an overall assessment.

Several reference levels can be defined for each indicator, which determine a certain number of performance intervals. Then, with the normalisation, all the indicators are conducted on a common scale, which is easily interpretable. The final score is not only a number, but also an informative measure of the problem assessed.

Another peculiarity concerns the possibility of building composite indicators for different compensation levels. In the MRP-WSCI methodology, this is possible thanks to two different indicators: Weak Composite Indicator (WCI), which follows a completely compensatory scheme, and the Strong Composite Indicator (SCI), which is completely non-compensatory. In MRP-PCI scheme, the Partially Composite Indicator (PCI) is built with the use of a different compensation index for each indicator, goal, and dimension of the indicator system. To the best of the authors’ knowledge, sustainability has not been assessed before taking into account all these issues jointly.

The rationality and flexibility of the method lies in considering the possibility that a Decision Maker believes that the compensations may not be the same at each level.

In particular, this methodology gives us the possibility to get a picture of the overall situation and at the same time to go back to the worst indicator. Highlighting weaknesses and providing very important information about possible interventions, this approach helps decision makers to define the improvement actions to be implemented.

Policymakers can better explore MCDM applications to prioritise projects and programmes to achieve SDGs and define public policies for the implementation of Agenda 2030 in different contexts. In addition, operators within different sectors of public and private organisations can better replicate and use existing MCDM models to improve their strategic decision-making processes regarding resource allocation to strategy companies associated with one or more objectives. (Sousa et al., 2021).

This methodology offers a great advantage in terms of the transparency and traceability of the method, making it easier to read and interpret the results. This final characteristic is crucial in the context of the 2030 Agenda, as the Sustainable Development Goals can be achieved with collaboration amongst all parts of society and not just from the political authorities. The other parties in society must have a clearer vision of the situation. In fact, just through a shared common ethic, the SDGs can be achieved.

## Conclusions

In this paper, two versions of the Multi Reference Point method were used and compared for assessing the sustainability of EU States, in the framework of the 2030 Agenda: the Multi Reference Point based weak and strong composite indicators (MRP-WSCI) method and the Multi Reference Point based partially compensatory indicator (MRP-PCI).

The analysis carried out of the objectives of Agenda 2030 for the European Union member states has shown a situation with evident difficulties from the point of view of social sustainability, with there still being major inequalities for economic and environmental sustainability, which stagnates in terms of sufficiency levels.

In general, Nordic countries have better performances, including those of the Scandinavian peninsula with Sweden in first place, followed by Denmark and Finland; the states of France and Austria can also be considered good. On the contrary, the States of Eastern Europe, including Romania, Bulgaria, and Greece, with insufficient values, achieve the worst levels. The development model of these three states should be completely reshaped in a sustainable way. Furthermore, the achievement of the objectives requires a continuous dialogue between all those entities directly and indirectly involved. Sustainable development depends on the coherence between the development policies of beneficiaries and development assistance providers, but achieving coherence is difficult and requires a great deal of cooperation between all stakeholders.

The performances of the remaining states do not reach values that can be considered good, but do not even constitute a bad situation in terms of global sustainability; they have some problems just in specific Goals or indicators, as shown by the analysis carried out.

As we have pointed out, many countries among those with the best global sustainability results have ranked worse in the environmental dimension. Furthermore, there are major differences in values between these states and the worst ones in this sphere, compared to the economic and social ones and this may be for several reasons.

Furthermore, in terms of the indicators, there is a tendency to give priority to social and development indicators and to pay less attention to environmental ones. Moreover, countries are not willing and/or able to collect data on the natural resources necessary to make possible a national or global assessment of sustainability. In the selection of environmental indicators, we also experienced this issue of the lack of available data.

The results obtained show once again how economic development takes place at the expense of environmental protection, leading us to wonder whether the SDGs can really be achieved and if development which is sustainable can become a reality.

This is the greatest challenge that humanity has ever had to face, as it encompasses complex problems which are all focused on science, without there being sufficient scientific knowledge spread throughout the world.

The 2030 Agenda can be a tool for guiding the change that we need: working collectively, involving countries and communities towards a common goal. To this end, the plan must adapt to reality and translate it into an ambitious sustainable development strategy, developed by all the territories and political, economic and social actors in a climate of collaboration with civil society too. By analysing the resources available and looking at specific objectives, we must define a clear roadmap for a transformation towards a fairer and more sustainable society, which can improve its social, economic and environmental footprint in the world. In this context, studies such as the present one makes it possible to establish a benchmark line, to specifically measure where we stand with respect to the achievement of global objectives, and the road that is still to be followed.

Nowadays, this is even more important when the challenges facing the world, in particular economic growth, poverty, inequalities, food and financial security, have been exacerbated by the Sars-Covid 2019 pandemic. For any future study, it would be interesting to analyse whether there is indeed a risk that the process of achieving SDGs will be even slower given the current situation.

## Abbreviations

DEA: Data envelopment analysis
MCDM: Multiple criteria decision making
MDGs: Millennium development goals
MRP-PCI: Multiple reference point partially composite indicators
MRP-WSCI: Multiple reference point weak-strong composite indicators
PCI: Partially composite indicator
SCI: Strong composite indicator (SCI)
S-W-W: Strong-weak-weak composite indicator
SDGs: Sustainable development goals
UN: United Nations
WCI: Weak composite indicator (WCI)
W-W-W: Weak-weak-weak composite indicator

## Footnotes

1 EUROPE 2020 A strategy for smart, sustainable and inclusive growth. Available at: https://eur-lex.europa.eu/legal-content/en/ALL/?uri=CELEX%3A52010DC2020.

2 Available at: https://ec.europa.eu/info/sites/info/files/political-guidelines-next-commission_en_0.pdf.

3 Available at: https://ec.europa.eu/commission/presscorner/detail/en/IP_18_3885.
